# Endobronchial Extranodal Marginal Zone B-Cell Lymphoma with Plasmacytic Differentiation

**DOI:** 10.7759/cureus.13104

**Published:** 2021-02-03

**Authors:** Alejandra Yu Lee-Mateus, Juan C Garcia-Saucedo, David Abia-Trujillo, Andras Khoor, Sabastian Fernandez-Bussy

**Affiliations:** 1 Pulmonary Medicine, Mayo Clinic, Jacksonville, USA; 2 Pulmonary and Critical Care Medicine, Mayo Clinic, Jacksonville, USA; 3 Pathology and Laboratory Medicine, Mayo Clinic, Jacksonville, USA

**Keywords:** endobronchial tumor, bronchoscopy, plasmacytic differentiation, non-hodgkins lymphoma

## Abstract

Endobronchial tumors (ET) are unusual and mostly malignant, presenting with non-specific symptoms that often delay appropriate diagnosis and treatment. Lymphomas in the airway represent less than 1% of pulmonary malignancies and require multidisciplinary approach for their management. We present a case of a 48-year-old male former smoker with a one-year history of recurrent respiratory infections and new-onset shortness of breath. Diagnostic tests included a chest computed tomography (CT) reporting the presence of an endobronchial mass and neck and cervical lymph node biopsies with inconclusive results. Bronchoscopy was successfully performed for tumor resection, improving the patient’s respiratory symptoms. Histological analysis described an extranodal marginal zone B-cell lymphoma (ENMZL) with plasmacytic differentiation; a subtype of non-Hodgkin’s lymphoma (NHL) in mucosa-associated lymphoid tissue (MALT), rarely found as an endobronchial growth. ET should be considered in the setting of persistent and worsening respiratory symptoms. ENMZL with plasmacytic differentiation is rarely found as an ET and diagnosis requires bronchoscopic intervention and extensive immunohistochemical analysis.

## Introduction

Endobronchial tumors (ET) are uncommon and represent 0.4% of all body tumors [1]. In most cases, they present with non-specific symptoms, such as cough, dyspnea, and wheezing, that resemble other infectious or chronic pulmonary disorders [2,3]. This poses a challenging scenario for the timely diagnosis and management of ET.

Airway lymphomas arise as a rare cause of endobronchial growth, with a predilection for Hodgkin's lymphoma over non-Hodgkin's lymphoma (NHL). NHL accounts for less than 1% of pulmonary malignancies, and its diverse pathological presentations often elude a clear diagnosis [4]. We report an unusual case of an endobronchial extranodal marginal zone B-cell lymphoma (ENMZL) with plasmacytic differentiation in the left mainstem bronchus resected with electrocautery and argon plasma coagulation (APC).

## Case presentation

A 48-year-old former smoker male (seven pack-year history) was referred to our clinic for evaluation of an endobronchial mass found on a chest computed tomography (CT). He presented with a one-year history of cough, myalgias, arthralgias, recurrent respiratory tract infections, and new-onset shortness of breath, with no fevers, weight loss, or night sweats. His previous workup included a non-diagnostic bronchoscopy and CT-guided neck lymph node and bone marrow biopsies with inconclusive results.

**Figure 1:**
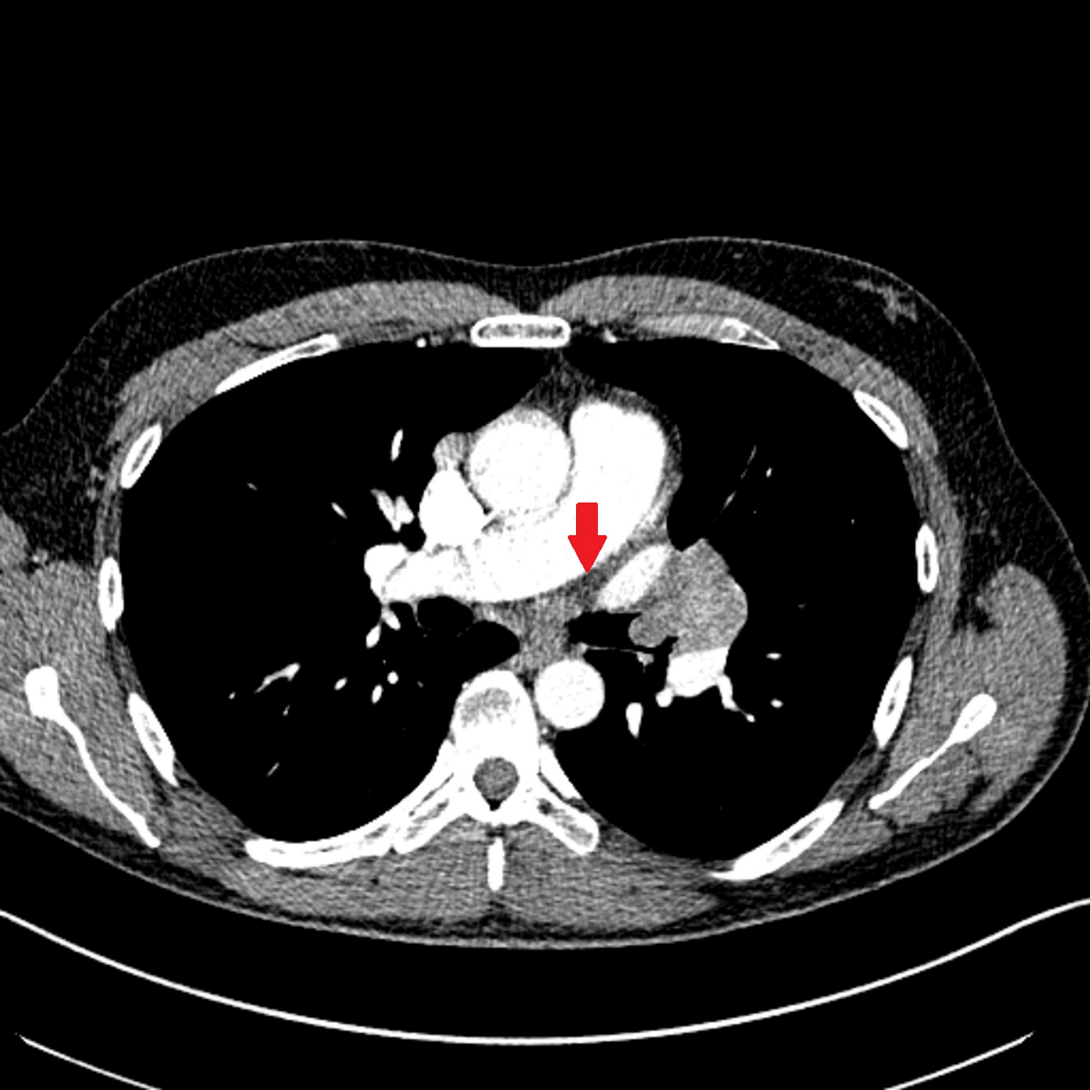
Chest computed tomography Chest computed tomography showing endobronchial mass compromising left upper lobe (red arrow).

Once in our care, a repeated chest CT scan confirmed the presence of an endobronchial mass occluding the left upper lobe with air trapping throughout the left lung (Figure 1). Bulky hilar and mediastinal lymphadenopathy was also noted. Pulmonary function tests showed a forced vital capacity (FVC) of 2.41 L (57%), forced expiratory volume in 1 second (FEV1) of 2.10 L (62%), and FEV1/FVC ratio of 87.3%. Compared to results reported one year ago, the FVC and the FEV1 decreased by 30% and 53%, respectively, showing signs of airway compromise.

**Figure 2:**
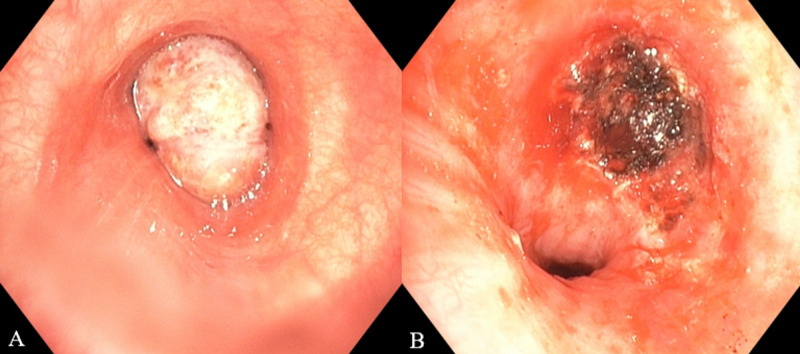
Bronchoscopy resection and argon-plasma coagulation (A) Flexible bronchoscopy showed a 1.5 cm endobronchial tumor in the distal left mainstem bronchus occluding 90% of the lumen. (B) Post-tumor resection achieving airway patency.

A new flexible bronchoscopy demonstrated an ET in the distal left mainstem bronchus, occluding 90% of the lumen (Figures 2A-2B). We debulked the tumor using electrocautery snare and APC, achieving a complete opening of the left mainstem bronchus. The rest of the airway was patent, and no immediate complications were noted.

A thorough review of the bone marrow biopsy reported a normocellular bone marrow and aspirate. However, the neck lymph node biopsy was positive for immunohistochemical stains cluster of differentiation (CD)20 and CD79a, highlighting clusters of B-cells, and CD138, kappa, and lambda showed increased atypical plasma cells with kappa restrictions. CD30, CD15, and paired box gene 5 (PAX5) were negative for Hodgkin-Reed-Sternberg cells, and Congo red stains were also negative for amyloid. Molecular studies for B-cell gene rearrangement identified a clonal rearrangement, supporting a B-cell disorder diagnosis.

**Figure 3:**
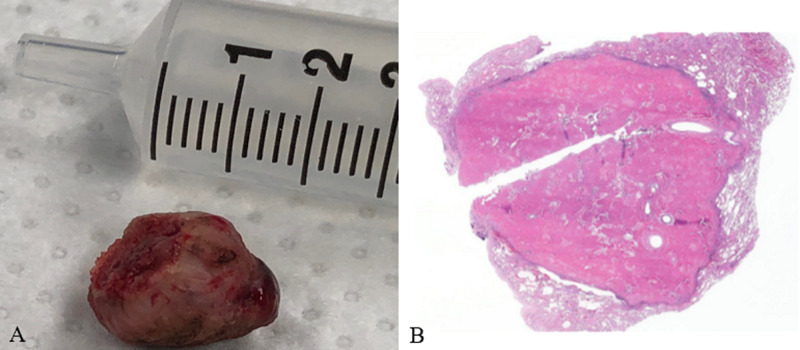
Histological analysis (A) Resected endobronchial tumor. (B) Histologic sections showed a 1.5 cm nodule composed of amorphous eosinophilic material and surrounded by clusters of lymphocytes and plasma cells. The eosinophilic material was reminiscent of amyloid, but negative for Congo red stain, suggestive of non-amyloid light chain deposition.

The histological analysis of the 1.5 cm endobronchial nodule described a polypoid tissue with neoplastic kappa light chain-restricted plasma cell infiltrates associated with amorphous eosinophilic material and surrounded by clusters of lymphocytes (Figure 3). Congo red stain was negative for amyloid. These findings were suggestive of non-amyloid light chain deposition.

Finally, mass spectrometry confirmed the predominance of kappa immunoglobulin light chains and did not identify any amyloid proteins in both the neck lymph node and the ET. In combination with the B-cell lymphoproliferative disorder, the patient was diagnosed with ENMZL with plasmacytic differentiation, a rare NHL of mucosa-associated lymphoid tissue (MALT) overlapping between a low-grade lymphoma and a plasma cell dyscrasia.

Treatment for ENZML comprises six courses of combination chemotherapy with cyclophosphamide, bortezomib, dexamethasone, and rituximab and follow-up by the Department of Hematology and Oncology at our institution.

## Discussion

ETs are an unusual finding, accounting for 0.6% of all pulmonary tumors. The majority of ET are malignant in adults, while benign lesions are more common in pediatric populations. Squamous cell carcinomas and adenoid cystic carcinomas comprise around two-thirds of malignant tumors in the tracheobronchial tree, followed by lymphoma, mucoepidermoid carcinomas, adenocarcinomas, and plasmacytomas [5,6].

Thoracic involvement presents in up to half of patients with NHL. However, pulmonary compromise is only seen in 3.6% and usually affects hilar and mediastinal lymph nodes, or lung parenchyma [4]. ENMZL of MALT is a subtype of NHL that arises at extranodal sites such as the gastrointestinal tract, salivary glands, lungs, skin, and orbit. The tracheobronchial tree is usually spared [7]. In fact, in a 75-case study of non-gastric MALT lymphoma, Zinzani et al. reported only one case of endobronchial lesion [8].

The most frequently described symptom is a slow-onset, mild, persistent cough that progresses to dyspnea. Constitutional B-symptoms (fever, night sweats, and weight loss) are less common, and in an eight-case review of endobronchial NHL, they were only recounted once. Our patient denied any constitutional symptoms, and aside from the shortness of breath due to airway obstruction, he remained in his usual health. Hoarseness, pleuritic pain, and hemoptysis have also been mentioned, and in the absence of airway obstruction, patients can remain asymptomatic [9]. However, the severity of symptoms is closely related to the obstruction progression and can rapidly evolve into respiratory failure [3].

CT and bronchoscopy are the initial diagnostic and therapeutic tools. Rigid and flexible bronchoscopy allows for direct visualization of the lesion, assessment of contiguous spread, and tissue sampling [2]. Based on these findings, precise diagnosis relays on histopathological, morphologic, immunophenotypic, genotypic, and molecular characteristics [5]. Bronchoscopic procedures such as tumor debulking, electrocautery, APC, and cryotherapy achieve reopening of airways and relief of respiratory symptoms.

ENMZL can present with overlapping manifestations of other blood cell lines. Plasmacytic differentiation occurs in approximately one-third of MALT lymphoma cases and includes light chain-restricted plasma cells located in the interfollicular and perifollicular regions or within colonized germinal centers. This differentiation is minimal in most cases, but can become aggressive, consisting of more than 80% of the total cellularity, as our patient presented [10]. Immunohistochemical stains CD20 and CD79a for B-lymphocytes, and CD138, kappa, and lambda for atypical plasma cells are positive in this mixed disorder. Congo red stain for amyloid and Hodgkin-Reed-Sternberg stains are negative [7], and mass spectrometry is usually required to confirm the diagnosis. Combination chemotherapy with cyclophosphamide, bortezomib, dexamethasone, and rituximab is the standard treatment with a favorable prognosis of over 80% five-year survival rates and mean survival rates surpassing 10 years [4,5]. 

## Conclusions

ETs are rare and usually present with non-specific respiratory symptoms. Malignancy is the more common presentation, requiring histological analysis for accurate subcharacterization. ENMZL is a subtype of NHL that can present with plasmacytic differentiation in up to one-third of cases. Management includes bronchoscopic intervention and chemotherapy, with a favorable prognosis.
